# The Dynamics of Visual Experience, an EEG Study of Subjective Pattern Formation

**DOI:** 10.1371/journal.pone.0030830

**Published:** 2012-01-26

**Authors:** Mark A. Elliott, Deirdre Twomey, Mark Glennon

**Affiliations:** 1 School of Psychology, National University of Ireland Galway, Galway, Republic of Ireland; 2 Department of Acoustic Design, Center for Applied Perceptual Research, Kyushu University, Fukuoka, Japan; City of Hope National Medical Center and Beckman Research Institute, United States of America

## Abstract

**Background:**

Since the origin of psychological science a number of studies have reported visual pattern formation in the absence of either physiological stimulation or direct visual-spatial references. Subjective patterns range from simple phosphenes to complex patterns but are highly specific and reported reliably across studies.

**Methodology/Principal Findings:**

Using independent-component analysis (ICA) we report a reduction in amplitude variance consistent with subjective-pattern formation in ventral posterior areas of the electroencephalogram (EEG). The EEG exhibits significantly increased power at delta/theta and gamma-frequencies (point and circle patterns) or a series of high-frequency harmonics of a delta oscillation (spiral patterns).

**Conclusions/Significance:**

Subjective-pattern formation may be described in a way entirely consistent with identical pattern formation in fluids or granular flows. In this manner, we propose subjective-pattern structure to be represented within a spatio-temporal lattice of harmonic oscillations which bind topographically organized visual-neuronal assemblies by virtue of low frequency modulation.

## Introduction

**Figure 1:**
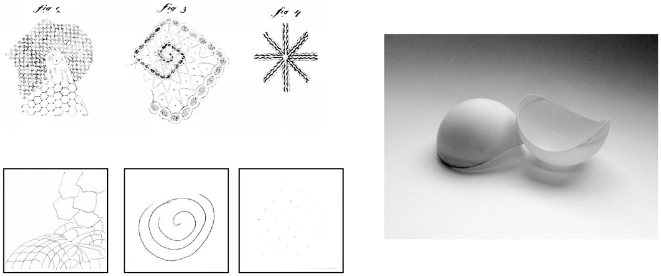
Sketches of Ganzfeld phenomena. Above: drawn by [1] these depict (right to left) “primary patterns”, “snail-rectangle” and “eight-beam”. Below: sketches from participants tested by Becker and Elliott (unpublished) of, (right to left) “hexagons”, “spirals” and “points”. The photograph shows goggles made from Ping-Pong balls (Reprinted from Consciousness and Cognition, Carsten Allefeld, Peter Pütz, Kristina Kastner, Jiří Wackermann, Flicker-light induced visual phenomena: Frequency dependence and specificity of whole percepts and percept features, in press/corrected proof, (2011), with permission from Elsevier).

Reports of purely subjective visual patterns are unique in spanning almost the entire history of psychological science. In his doctoral thesis, Jan Evangelista Purkinje (1787–1869) [1] describes the spontaneous appearance of lattice-like arrangements of rectangles as well as honeycombs and circular or semicircular forms alongside spiral type patterns or *Schneckenrechteck* (‘snail rectangle’)(Figure 1). In early studies, Purkinje's contemporary, Gustav Theodor Fechner and subsequently Benham described subjective impressions of color as well as of form on a spinning disk [2], [3]. Since the development of stroboscopic technologies the majority of subsequent studies have used intermittent photic stimulation, notably the so-called ‘flickering Ganzfeld’ in which the participant is exposed to flicker across the entire visual field [4], [5], [6], [7], [8], [9], [10], [11], [12], [13], [14], [15]. In the Ganzfeld, subjective experiences appear at delays of between several hundred milliseconds to several seconds from flicker onset [4], [14]; they appear localized in external space, appearing to occupy the center of the visual field [2] and range from simple phosphenes, colors and optical flow fields to spatially well-defined, complex kaleidoscopic visual patterns [7], [14]. Consistent with Purkinje, elementary hallucinations that include phosphene-type experiences as well as complex visual patterns, may comprise rectangular as well as circular forms, sometimes including rotating radials or spirals that give the impression of a tunnel [8]–[15]; other geometric forms, in particular honeycombs (hexagonal lattices) [7]–[15] are reported while the Ganzfeld may be divided by lines of different types (including zigzags and waves) [8]–[15] or filled with simple dots or points [8]–[11], [14], [15]. Patterns frequently transform within the Ganzfeld according to taxonomic relations [14] that appear to relate to form complexity [15]: radials will appear significantly more often than not within the same epoch as zigzags, spirals and lines, while points will appear in isolation and not with any other form [14]. Remarkably, there is very strong agreement between studies, between participants within studies and even at particular frequencies with respects to the type of subjective experience: a number of studies report appearance of exactly the same forms, with reports consistent across both repeated measures and participants, at flicker frequencies that differ with a precision of 1 Hz or less [13]–[15]. The appearance of spontaneous patterning in the static (non- flickering) Ganzfeld has also been reported as precursory to the appearance of more complex hallucinatory phenomena [16].

Because of the absence of a corresponding stimulus, Ganzfeld phenomena represent a problem for theories of perception concerned with ecological optics [17] and Gestalt grouping [18]. In the Ganzfeld, neither flow fields, nor patterns afford any particular behaviour; indeed, participants may experience mild akinesia and brain-response mechanisms such as focal attention are very difficult to deploy. Complex patterns are also easy to define in terms of spatial organization. This seems contrary to Gestalt theory in which form complexity is associated with the organizational principle of simplicity (the *minimum principle*) in which percepts will always be as ‘good’ as prevailing stimulus conditions allow [18]: in the Ganzfeld the prevailing conditions consist only flickering light. However, Gestalt theory also states that the brain acts dynamically to modify visual input towards good form [19]. While the brain was believed to assimilate or exaggerate the percept according to comparison with memories of similar forms, it was also believed capable of autonomous dynamics in which perceptual organization could be achieved even if there were no direct visual input.

Reconciliation of subjective visual patterns – as empirical phenomena – with perceptual theory thus seems possible in consideration of neural dynamics. However, a more precise set of hypothesis require consideration of exactly which type of neural dynamic accompanies subjective form perception, as well as where in the brain this dynamic might be found. Concerning location, fMRI recordings [6] indicate all classes of subjective Ganzfeld phenomena (i.e. color, patterns and optic flow) to be associated with an increased BOLD response in a variety of brain regions. In visual brain areas these include bilateral occipitotemporal regions centered on the fusiform gyrus and extending to lingual and inferior temporal gyri. In [6], apparently spontaneous (non-stimulus related) phase coherence was revealed in analysis of visual-evoked potential (VEP) recorded between occipitoparietal and central midline electrodes.

Complex visual forms are believed to be represented by synchronized neuronal oscillations at frequencies in the gamma band (30–100 Hz), particularly in visual brain areas [20], [21], [22], [23], [24], [25]. These oscillations are not usually phase locked to the stimulus [26] indicating their potential as a means for subjective patterning. We expected that the experience of subjective patterns would be associated with variation in the gamma component of the VEP, in particular activity under electrodes positioned over occipitotemporal regions. We also considered the potential relation between subjective pattern formation and the autonomous brain dynamics related to energy minimization [9]. Related to this, analysis of coherence intervals in the EEG beta to gamma bands (18–30 Hz) has revealed them to be of shorter duration and subject to less variance for stimuli with fewer grouping solutions [27]. This lead us to expect that the brain regions concerned with subjective pattern formation might exhibit a similarly reduced variance relative to activity recorded elsewhere on the scalp. Finally and considering the usually very high correspondence between the particular subjective forms reported by individual participants [13], [14], we also expected inter-participant variances in the VEP to be low, indicating the common representation to be associated with a common process.

## Results

### Subjective Reports

**Figure 2:**
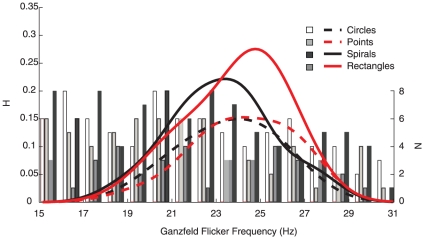
Histogram of subjective reports over flicker frequency and associated kernel-density estimates. As reported by [14] all reports were non-uniformly distributed over frequency indicating that trial-wise instruction to report a particular pattern on a particular trial did not confound with the normal tendency to experience patterns over a particular range of flicker frequencies. Lillifors tests failed to reject the Null Hypothesis of normality indicating reports “circles”, “spirals” and “rectangles” to be normally distributed (all p>.05 modes at 24, 23 and 25 Hz). Reports “points” were lognormally distributed with mode at 24 Hz.

Five participants were selected on the basis of pilot testing which identified individuals likely to report subjective phenomena on >50% of trials. These participants were then prompted to report the appearance of one of four forms: circles, points, spirals or rectangles in the flickering Ganzfeld. These forms were chosen because they show a common range of appearance over stimulating frequency [14] and appear independently, either of one another (spiral, rectangle), or relative to all other subjective forms (circle, points) [14]. In this way we aimed to ensure minimum confusability between what participants were required to report. On average, forms were reported on 54% of trials while the arcsine-transformed report percentages collapsed over stimulating frequency did not differ significantly between forms. Similar to previous reports, all form reports were non-uniformly distributed across the range of stimulating frequencies. Reports “Points” were lognormally distributed while other reports were normally distributed, appearing more frequently at frequencies centered on 23–25 Hz (Figure 2).

### Subjective form in the visual-evoked potential

**Figure 3:**
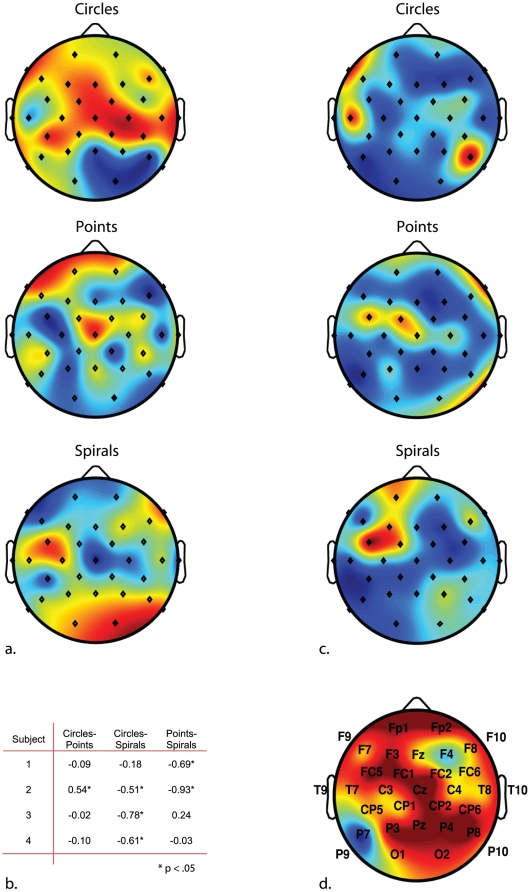
Scalp topographies of averaged component clusters. Illustrated separately in (a) are scalp maps for reports “circles”, “points” and “spirals”. For reports “rectangles” a cluster solution that included at least 4 of the 5 participants was not found and so this is not illustrated. In (b) a correlation matrix shows the relationship between the averaged power distributions on the scalp for each cluster. Interestingly, reports “Points” differs significantly from reports “spirals” indicating a different distribution of activation on the scalp associated with patterns of the highest and lowest dimensionality (see text for related discussions). In (c) the scalp topography of mean variances are illustrated separately for reports “circles”, “points” and “spirals”. For Figures 3(a) and 3(c) scales show red for highest to blue for lowest activation or variances, respectively. In (d) the scalp map represents the proportion of variances at each electrode site that are significantly lower than the average variance across the scalp (proportions calculated between participants, blue low – red high proportions). Consistent with previous neuroscientific data [4] as well as our expectations, variances were consistently lower at posterior ventral electrodes over occipitotemporal cortex. This indicates pattern formation results in a reduction of cortical activity, which is consistent with both Gestalt theory and recent neuroscientific findings [27].

High intensity flicker evokes a multichannel VEP that varies in different brain regions irrespective to the appearance or quality of subjective phenomena [28]. The VEP includes activity at frequencies corresponding to the stimulation frequency, the so-called *photic driving response*. It was not our aim to examine the photic driving response in detail as this does not relate directly to representation of subjective phenomena [2], [29]. However, this left us with the problem of how to identify the response that best describes subjective pattern representation. To solve this problem and as a first step we employed a series of independent component analyses (ICAs) on the time series of the averaged VEP, within participants and across reports. As a control, identical ICAs were conducted on the time series of the averaged VEP for trials upon which no target form had been reported. By virtue of blind source separation, ICA allowed us to isolate major sources of variance in the VEP with a minimum set of assumptions [30]. The result of this analysis included topographical power distributions which were subsequently clustered with the aim to classifying components according to topographical power distribution, separately for each of the patterns studied. Reports ‘rectangles’ (overall lower in frequency than the other reports Figure 2a) failed to cluster a minimum of 4 of the 5 participants' components and so were not analyzed further. For the other patterns, in each case one component cluster was found to satisfy our inclusion criteria (the averaged power distributions derived from these clusters are shown in Figure 3a). For the no-report trials, components failed to cluster across more than 3 subjects, while were there no clusters with a similar topographical power distribution to those illustrated in Figure 3a. On this basis we concluded that the scalp distributions accompanying subjective experience of circles, points and spirals corresponded to the process involved in representing those subjective forms, rather than being a general characteristic of the VEP to photic stimulation.

By default, our application of cluster analyses selected topographical power distributions with positive correlation between participants. Conversely, Figure 3b shows that the participants-wise correlations between the different patterns were, if significant, very largely negative, indicating that representation of the different patterns is associated with different topographical power distributions. However, one of our hypotheses concerned a common source of low variance in the cortical response rather than power distributions per-se. We tested this in the VEP by examining topographical distribution of variances between participants, separately for each pattern. Figure 3c shows the averaged between-participants variances derived from ICAs for points, circles and radials. Although there are clear similarities between variances under right posterior and left anterior electrodes, the overall patterns of variances did not correlate across subjective pattern. Consequently, analyses aiming to identify variances significantly lower than the mean variance were conducted separately for each set of pattern components.

For this analysis, we initially considered the possibility that noise in the form of particularly high variances (e.g. under right fronto-central as well as right anterior and left posterior sites) might skew our estimate of mean variance and its associated standard error. To compensate we replaced variances higher than the upper 99.995% confidence intervals (CI, adjustment correcting for multiple comparisons) with the average of the residual variances. We then recalculated the upper and lower CIs from the adjusted distributions using z-test analysis. Consistent with our hypothesis, this analysis revealed variances to be lower than the lower 99.995% CI at electrode P7, lying over occipitotemporal cortex. Unexpectedly, this was the only electrode at which variances were estimated to be lower than the adjusted CI for all subjective patterns, although this offers very strong corroboration of our analysis according to previous fMRI and EEG data in which subjective pattern representation correlates with brain activation in occipitotemporal cortex [4]. This analysis also indicates a consistent and theoretically valid measure of subjective-pattern representation derives from analysis of the VEP power variances. Relative to [4] and [27], the unilateral left hemisphere reduction in variance might come about due to asymmetries in event-structure timing between hemispheres favoring left rather than right hemisphere [31], [32].

### Dynamic representation of subjective patterns

**Figure 4:**
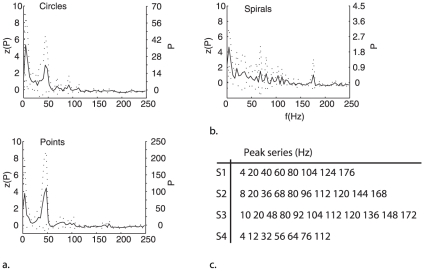
Frequency components derived from discrete Fourier analysis of the averaged component time series. The left y-axis gives normalized power while the right y-axis gives actual power. The dotted lines indicate standard errors. In (a) analysis of circles and points reveals peaks at theta (6 Hz) and mid gamma-band (46 and 48 Hz) frequencies. Subjectively, both points and circles appear at a set of independent loci that are apparently randomly distributed across the visual field. They are characterized by particularly high power in both theta and gamma bands which may index an as yet unresolved process of pattern completion. In (b), and by contrast, spirals refer to a spatially contiguous and so relatively well defined visual pattern. Fourier analysis reveals these reports to be accompanied by very low power distributed across multiple peak frequencies; including a major peak at 4 Hz. Fourier analysis carried out on the component time series for reports “spirals” for each participant separately reveals power spectra with multiple peaks tabled in (c). All peaks bar one are multiples (harmonics) of a fundamental frequency of 4 Hz, indicating subjective experience of spirals to be accompanied by a lattice of harmonic activity in the EEG.

We expected that the experience of subjective patterns would be associated with variation in cortical gamma-band activity and so subsequent Fourier analyses were carried out on the averaged time series associated with clustered power distributions (reconstructed from the VEP by the ICA). The normalized frequency components are shown in Figures 4a and 4b. The appearance of all patterns is associated with an increase in low frequency power in the range 4–6 Hz. These frequencies may modulate the phase of the photic driving response to enable selection of an appropriate frequency response mode [33]. In the case of circles and points, the peak response frequencies are at 46 and 48 Hz, frequencies within the gamma band that are close to the first harmonic of the mean driving frequencies (Figure 2). However for spirals there is no unimodal response frequency and instead several modes are evident across the range of sampling frequencies, including frequencies lower and higher than the gamma band (Figure 4b).

The overall power of the peak gamma response to points was considerably greater than that to circles (Figure 4a) although the presence of unimodal gamma peaks in both cases may be similarly explained in terms of the nature of the subjective experience: both points and circles involved the appearance at and/or extension of multiple independent positions (or loci) in the Ganzfeld. However, in neither case did these loci connect to form a spatially extended pattern, suggesting that high power in the gamma band here represents the continuation of process of perceptual organization in which a solution pattern is yet to be resolved [27]. For spirals, a pattern emerges that comprises either a phenomenally continuous or discontinuous (as in the case of Purkinje's example, Figure 1) but nonetheless connects a series of Ganzfeld loci. Connectivity also characterizes the distribution of peak frequencies in the Fourier analysis: a participants-wise examination of peak frequencies (Figure 4c) reveals ^35^/_36_ peaks to be located at precise harmonics of a fundamental frequency of 4 Hz. This analysis suggests spiral patterns emerge in the context of a coherence lattice of correlated frequencies related to a fundamental delta frequency. Consistent with recent theory [33] we assume the delta-frequency response ensures global stability and as a result the subjective binding of Ganzfeld loci. This may be achieved by an adjustment of all relevant local neuronal responses to occupy a position in the phase lattice that is harmonically related to the fundamental.

## Discussion

Subjective patterns are dynamic phenomena that are not only generated in the brain. Clues to their origins may be deduced from identical pattern formation in other modes. For instance, there is direct equivalence between the arrangement of subjective patterns in the brain and patterns formed in fluids and granular layers or flows [34], [35]. The surface of a fluid is an extended dynamical system for which the natural variables are the amplitudes and phases of the wavelike deformations. Such systems are complex and in the case of fluids, pattern emergence is a direct consequence of the underlying instabilities of the system: systems that are linearly unstable lead to divergent response functions with several possible solutions. By analogy we may consider the surface of the fluid to be equivalent to the organization of neurons in the brain and thus explain several aspects of subjective pattern formation. For instance, the evolving and often mutually exclusive emergence of subjective patterns [14] suggests this occurs as a consequence of very similar instabilities to those found in fluids. In fluids, when wave amplitudes are large, the nonlinear effects lead to chaotic dynamics and high dimensionality. This corresponds to the combination of relatively large amplitude oscillations at gamma and delta/theta frequencies, which accompany the emergence of distributions of points or circles in the Ganzfeld. Because they include multiple independent loci, both points and circles are relatively high dimensional patterns as compared with spirals. Spirals are associated with a lattice of low amplitude harmonic oscillations which corresponds to a stable solution and therefore low dimensionality. In fluids, what emerge under similar conditions are regular patterns such as hexagons, squares and stripes, which are remarkably similar to patterns very frequently reported in the flickering Ganzfeld. They also emerge in fluids at similar frequencies to those recorded in the present study (1–120 Hz) [1]–[15], [35]–[37]. The amplitude and phase of fluid waveforms depend on state parameters such as fluid viscosity, driving frequency and acceleration. Subjective patterns relate to at least two of these variables: the non-uniform distribution of reports of subjective patterns over flicker frequency indicates driving frequency to be of significance. We believe driving frequency interacts in phase with spontaneous brain rhythms and, because oscillatory behavior is generally described as a flow on a limit cycle (or closed loop), the anatomical distribution (i.e. viscosity) of contributive neuronal systems are the second variable of interest.

We propose a combined harmonic/topographical lattice in which connected patterns such as spirals are directly analogous to the topographical distribution of neurons, temporally bound by virtue of common (sometimes harmonic) alignment in phase. This means that experience of particular pattern structure is directly dependent upon the structure of the lattice. However topographically, the structure of the lattice may be complex and not directly isomorphic with the pattern structure – if evidence for topographic mapping from retinal coordinates to cortical coordinates is of relevance ([38], p.128). In this mapping a point (r, Θ) in polar coordinates on the retina is mapped to (log r, Θ) in Cartesian coordinates in the cortex. In terms of spatial or topographical representation this would mean that the spiral pattern reported here would be represented by a pattern of synchronization spatially diagonal to the retinotopically preserved and layered organization of visual cortex.

Representation of such a pattern would thus require the synchronization of a number of neurons with both adjacent and non-adjacent receptive fields that are located across visual-cortical layers and across neurons representing different retinotopic coordinates. With this diagonal structure in mind, a synchronization lattice exploiting temporal phase, that is approximately harmonic rather than a non-linear may in fact provide a relatively simple solution to bind contributive neurons to a single representational structure. In addition, and given this subjective pattern occupies apperceptive space beyond that normally responding to the retinal fovea, we might also expect the harmonic/topographical lattice to accommodate differences in the timing of neural systems responding to foveal and peripheral retinal input. Differentiation of the pattern into a lattice of harmonics might serve to maintain binding across the subjective pattern independent to any difference in neuronal response latencies and we might expect neurons responding to pattern loci closer to the center of the visual field to map to lower frequencies with neurons at more peripheral loci responding at higher frequencies. Given oscillatory activity at very high frequencies in laminar thalamocortical networks [39] it is in principle possible that a spatially extensive pattern could become represented by oscillations over a relatively broad band, including frequencies in excess of 100 Hz.

**Figure 5:**
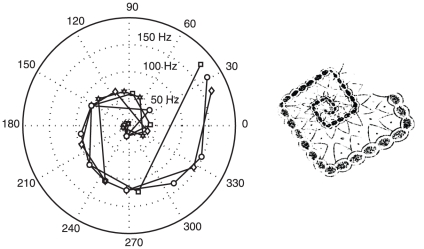
Representations of Spirals. The figure to the left illustrates the outcome of participants-wise Fourier analyses of the spirals components time-series in which the sets of harmonic response frequencies for each participant (tabled in Figure 4c) are plotted in circular coordinates over their square root. Low frequencies plot to the center of the polar plot, with higher frequencies plotted at increasing distance from the center. The individual participants' data are represented by different symbols. Each harmonic series plots spiral patterns in a system of polar coordinates that models the circular phenomenal region of the Ganzfeld. Regular patterning in circular coordinate space of this type is not achievable with regularly spaced number series or random number series derived from a uniform distribution. To the right and by analogy, the figure depicts Purkinje's drawing of a spiral-like subjective pattern.

Figure 5 offers some evidence to suggest this is the case. The Figure illustrates a representation of the participants-wise Fourier analyses of the spirals components time-series. In this representation the sets of harmonic response frequencies for each participant (Figure 4c) may be plotted as spiral patterns in the system of polar coordinates that models the circular phenomenal region of the Ganzfeld. This is remarkable but it is not an altogether surprising outcome because subjective patterns will be generated by the spatio-temporal lattice within which they emerge in the brain. However, that they appear to participants not as a single diagonal stripe but as a spiral (in other words faithful to retinotopic coordinates) extends the idea of mind-brain isomorphism as proposed by Gestalt theorists [18], [19] to include the idea that the brain interprets its own re-representation of visual structure in a meaningful and prospectively an ecologically valid fashion.

The flickering Ganzfeld has been described as a means of turning off perception and allowing access to a natural representational template resulting from brain dynamics alone [2]. Our analysis argues that the conscious states that arise from these templates are not only a brain property but a property of brain states as complex systems. Accordingly, we support recent calls to adopt a formal modeling approach to subjective phenomena [15], but in so doing we emphasize the use of neuroscientific data as variables. In this way, and perhaps only in this way we will be able to provide a very precise mathematical description of the spatio-temporal lattices created by dynamical systems in the brain that is directly analogous with the patterning of conscious states.

## Methods

### Ethics Statement

The study was approved by a Research Ethics Committee convened by the School of Psychology at NUI Galway and was conducted according to the principles expressed in the Declaration of Helsinki. Written informed consent was obtained from all participants involved in the study.

### Participants

Fifteen healthy volunteers (3 male, mean age 21.7 years, normal or corrected to normal vision) gave written informed consent to their participation in the study. They had no prior history of neurological, neuroleptic or psychiatric disorders and were free to discontinue the experiment at any time.

The study consisted of two phases. The purpose of the first, screening phase (twenty minutes in flickering Ganzfeld conditions) was to select participants likely to experience and to be able to report subjective forms. Those who hallucinated on >50% of trials were asked to participate in phase two of the experiment. In the second phase, an EEG was recorded from eight participants as they completed a free-report paradigm in flickering Ganzfeld conditions. The data of three participants was excluded due to excessive ocular and/or muscular artefacts. Thus the final EEG sample for analysis consisted of five participants (1 male, mean age 22.4 years, normal or corrected to normal vision).

### Apparatus and Stimuli

Flickering light was presented using a specially constructed device consisting a cluster of four LEDs (Type NSPW315DS, 3 mm white light emitting diodes with 60° radiation angle, 20 mA 3.2 V, 3400 mcd, Conrad Electronic GmbH, 92240 Hirschau, Germany). Participants wore anatomically shaped ping-pong ball halves, applied over the eye cavities to create an almost perfectly homogenous visual field. During stimulation, a visual Ganzfeld was produced by rapid and intermittent square-wave light pulses of 3,000 cd/m^2^ emitted simultaneously from each of the four diodes. Stimulus generation and response collection were ensured by a PC running under MSDOS and generated in the C programming language. The precise temporal delivery of light pulses was achieved using PCI technology timer card (CIO-CTR05 with CTS9513 chip capable of temporal resolutions of up to 5 kHz) mounted in a conventional IBM compatible PC running under MSDOS and connected to the four LEDs. The experimental machine was connected to a second machine dedicated to EEG data analysis via parallel port for the purpose of stimulus- and response-event triggering.

### Design and Procedure

Participants sat facing the diode cluster at a distance of 1 meter and were required to press a response key with the left index finger as quickly as possible on initially experiencing a target subjective pattern. A specific target pattern (circle, point, spiral and rectangle) was announced to observers verbally and immediately in advance of trial onset. In the case of a button press the flicker presentation terminated. If the observer did not experience the target subjective pattern, the trial was allowed to time out after 30 seconds and a zero response was recorded. Sixteen flicker frequencies were employed in the range 15–30 Hz at which each of the target subjective patterns had been reported previously with significantly greater than zero probability [14]. Each participant was prompted for each pattern twice and thus completed 128 trials overall. Both the presentation order of flicker frequency and the requested target pattern were varied pseudo-randomly across the 128 trials for each participant.

### EEG data acquisition

EEG signals were recorded and digitalized using an EEG amplifier (QuickAmp-40, Brain Products GmbH, Munich). Electrophysiological activity was referenced to the common average of all channels and data were sampled at 250 Hz and analogue-filtered via a 0.15 high-pass filter and a 100 Hz low-pass filter. Additionally a notch filter at 50 Hz was applied. EEG was recorded from 30 Ag/AgCl scalp electrodes arranged according to the extended 10–20 system and mounted on an elastic cap (EASY CAP EC40, EASYCAP GmbH, Herrsching-Breirbrun, Germany): electrodes were, Fp1, Fp2, F7, F3, Fz, F4, F8, FC1, FCz, Fc2, Fc5, Fc6, Tp9, C3, Cz, C4, TP10, CP5, CP1, CP2, CP6, T7, P3, Pz, P4, T8, P09, PO10, O1, O2. Impedances were kept below 5 kΩ. Ocular activity was measured via EOG channels mounted at the outer canthi of the right and left eyes, and approximately 3 cm above and below the right eye, respectively.

### EEG data analyses

Individual participant data were initially inspected by three raters (MAE, DT and MG) utilizing the raw data inspection transformation implemented in BrainVision Analyzer 2.0 (Brain Products GmbH, Munich) Data were excluded from analysis if no target form was reported. The remaining data (on average 69 trials per participant) were segmented into epochs of 2,100 ms with reference to response (i.e. 2,000 ms in advance to 100 ms after stimulus trigger). Following this, an eye movement correction procedure was implemented using the ocular correction algorithm implemented in the Brain Vision Analyzer software (also described in [40]). Data epochs were then subject to analysis using an ICA with infomax algorithm [30], undertaken on averaged VEP for each participant and each subjective form, separately. Subsequent classification of the independent components was undertaken using cluster analysis. This analysis calculated the Euclidean distance between variations in amplitude across the scalp as reconstructed from the VEP by the ICA. The cluster analysis computed linkages in a hierarchical cluster tree based on the average distances between component activations. This analysis was set to divide the data, overall, into 10 clusters with reference to the cophenetic correlation coefficient. For a 10 cluster solution, this was calculated at 0.86, indicating this solution to be the most accurate representation of the original data. Clusters were considered for further analysis if they included data from more than 80%, or 4 of 5 participants and on this criterion clusters were identified for each of circles, points and spirals. These are described in Figure 3 and the main body of text. Subsequent 256-point Fourier analyses were carried out across component time series. This analysis was carried out cluster-wise, but separately for each time series with the resulting power distributions averaged and presented in Figure 4. . ICA and cluster analyses were undertaken using Matlab 2010 Signal Processing and Statistics toolboxes alongside ICA algorithms available in the EEGLAB 5.03 toolbox [41].
